# Combining membrane potential and calcium imaging in brain slices using the voltage-sensitive dye ElectroFluor630 and the calcium indicator Calbryte520

**DOI:** 10.1117/1.NPh.13.S2.S23206

**Published:** 2026-05-13

**Authors:** Shirin Ghasemiform, Marco Canepari

**Affiliations:** a Université Grenoble Alpes, CNRS, LIPhy, Grenoble, France; b Institut National de la Santé et Recherche Médicale, Paris, France

**Keywords:** voltage imaging, calcium imaging, CMOS cameras, brain slices, electrical activity, hippocampus

## Abstract

**Significance:**

Wide-field imaging from brain slices stained with a voltage-sensitive dye (VSD) and simultaneously loaded with a ${\mathrm{Ca}}^{2+}$ indicator allows investigating neuronal excitability and synaptic transmission at a multi-cellular scale. So far, achieving this combined imaging has been limited by experimental constraints.

**Aim:**

We assessed the ability of the red-IR emitting VSD ElectroFluor630 (EF-630) to be combined with blue-excitable green-emitting ${\mathrm{Ca}}^{2+}$ indicators to record signals elicited by electrical stimulation in hippocampal slices.

**Approach:**

Transversal mouse hippocampal slices were stained with EF-630. ${\mathrm{Ca}}^{2+}$ indicators, either Fluo-4, Fluo-8, Cal520, or Calbryte520, were loaded using their AM-ester forms. Fluorescence, during stimulation of the CA3 region, was imaged at 5 kHz from the hippocampal areas of $∼750\times 250\;{\mu m}^{2}$ at 1-μm pixel resolution.

**Results:**

After assessing all ${\mathrm{Ca}}^{2+}$ indicators, we selected Calbryte520 for achieving >30-min stable recordings in combination with EF-630. Action potentials and related ${\mathrm{Ca}}^{2+}$ transients were sequentially detected in the CA3 stimulated area, whereas synaptic signals were observed in the CA1 region. On these signals, we tested the pharmacological blockade of either action potentials or glutamatergic synaptic potentials.

**Conclusions:**

We report unprecedented optical measurements of both electrical and ${\mathrm{Ca}}^{2+}$ transients in brain slices, providing unique information on neuronal excitability and network activity.

## 1 Introduction

Membrane potential ($V_{m}$) imaging in brain slices using a voltage-sensitive dye (VSD)^1^^,^^2^ allows monitoring electrical neuronal activity from small populations of cells, enabling investigation of both neuronal excitability and synaptic transmission. Separately, ${\mathrm{Ca}}^{2+}$ imaging in brain slices loaded with AM-ester dyes^3^^,^^4^ allows measuring transients of intracellular ${\mathrm{Ca}}^{2+}$ concentrations that can be related, in different manners, to the electrical activity. Thus, complementary information obtained by combining the two optical recordings might permit deep understanding of network activity. $V_{m}$ imaging has been performed in slices from several brain areas including the cortex,^5^^–^^11^ the hippocampus,^12^^–^^16^ the olfactory bulb,^17^ and the cerebellum,^18^ with various absorbance or fluorescence VSDs^19^ including di-4-ANEPPS, RH795, RH414, RH479, RH155, and RH482. Using these dyes, slices were stained for several minutes, and fluorescence changes ($\Delta F/F_{0}$) associated with $V_{m}$ transients were in the range of 0.05% to 0.5%, requiring the collection of large numbers of photons to be distinguished from photon “shot” noise. This was normally achieved using fast photodiode arrays, at the cost of lowering spatial resolution, and only recently using complementary metal-oxide-semiconductor (CMOS) cameras with higher spatial resolution.^20^^,^^21^ Regarding ${\mathrm{Ca}}^{2+}$ imaging in brain slices, this was done using several high-affinity indicators, in particular probes with fluorescence excited by blue wavelengths and emitting green wavelengths (e.g., “fluorescein”), such as Calcium Green,^22^ Oregon Green BAPTA-1,^23^ Fluo-3,^24^^,^^25^ or Fluo-4.^26^^–^^28^ ${\mathrm{Ca}}^{2+}$ activity was recorded after incubation of brain slices with the AM-ester forms of ${\mathrm{Ca}}^{2+}$ indicators normally for >30  min. In principle, $V_{m}$ and ${\mathrm{Ca}}^{2+}$ imaging can be combined in the same preparation if the fluorescence spectra of the two dyes are distinct. In practice, the ability of $V_{m}$ and ${\mathrm{Ca}}^{2+}$ imaging to explore neuronal excitability and synaptic transmission, as well as to combine the two techniques, is constrained by several technical aspects which are listed below.

1. The efficiency of both VSDs and ${\mathrm{Ca}}^{2+}$ indicators to stain (or load) neurons must be adequate, and the amplitude of the resulting signals ($\Delta F/F_{0}$) must be large enough to produce measurements with sufficient signal-to-noise ratio (SNR). Optical signal quality, therefore, depends on the solubility of VSDs in water and on the penetration of AM-ester forms into cells, as well as on the efficiency of esterases to cleave the AM group releasing the indicator into the cytosol.
2. Biological experiments require dye stability to enable recordings for long periods. This is given by the VSD retention in the plasma membrane, or ${\mathrm{Ca}}^{2+}$ dye retention in the cytoplasm, as well as by the dye resistance to photodamage produced by light exposure.
3. For a combination of $V_{m}$ and ${\mathrm{Ca}}^{2+}$ imaging, ideally, there should be absolutely no emission of each dye in the wavelength recording window of the other one. In practice, the VSD RH414 was combined with Calcium Orange^29^^,^^30^ and the VSD Di-2-ANEPEQ with Calcium Green-1,^31^ but in both combinations, dyes had overlapping excitation. Thus, the ability of obtaining “clean” $V_{m}$ and ${\mathrm{Ca}}^{2+}$ signals relied entirely on each dye giving only a negligible component in the wavelength recording window of the other one. In studies with dyes injected into single cells, Di-2-ANEPEQ (JPW1114) and Oregon Green BAPTA5N had negligible crosstalk in recordings from CA1 hippocampal pyramidal neuron dendrites^32^^,^^33^ but not in the dendrites of cerebellar Purkinje neurons^34^ and of layer 5 neocortical pyramidal neurons.^35^ Thus, no overlapping excitation is also an ideal condition to always obtain independent $V_{m}$ and ${\mathrm{Ca}}^{2+}$ recordings.

Here, we report combined $V_{m}$ and ${\mathrm{Ca}}^{2+}$ imaging measurements that extend this type of measurement from the single-cell approach, where dyes are injected into single cells, to a population imaging approach where brain slices are stained/loaded with VSD and ${\mathrm{Ca}}^{2+}$ indicators. $V_{m}$ imaging was performed with the VSD Di-4-ANEQ(F)PTEA,^36^ also known as ElectroFluor630 (EF-630), which was used in cardiac preparations^37^ and only recently in brain slices.^38^ This indicator is soluble in water and can stain slices with ∼1  min of exposure at micromolar concentrations. By stimulating with an extracellular electrode, the synchronized action potential (AP) in the adjacent area produced an absolute fluorescence transient >1% when fluorescence was excited at 640 nm. The slower $V_{m}$ changes in other areas, associated with excitatory postsynaptic potentials (EPSPs) and asynchronous firing, could be as large as 0.5%. We assessed that these signals were stable for at least 30 min. As EF-630 was excited at 640 nm and emits at longer wavelengths, this VSD can be combined with common blue-excited and green-emitting ${\mathrm{Ca}}^{2+}$ indicators. Among these dyes, we assessed the performance of Fluo-4, Fluo-8, Cal520, and Calbryte520 and established that the last one provides large ($\Delta F/F_{0}>1\%$) signals with stability comparable to that of EF-630. The relatively high amplitude of both $V_{m}$ and ${\mathrm{Ca}}^{2+}$ signals allowed resolving them by acquiring images of ∼750×250  pixels at 5 kHz using a state-of-the-art CMOS camera. Thus, in a standard hippocampal preparation, we report an initial characterization of functional imaging signals obtained with the unprecedented combination of EF-630 and Calbryte520.

## 2 Materials and Methods

### 2.1 Ethical Approval

Experiments were performed at the Laboratory of Interdisciplinary Physics in Grenoble in accordance with European Directives 2010/63/UE on the care, welfare, and treatment of animals. Procedures were reviewed by the ethics committee affiliated to the animal facility of the university (E3842110001). Mice (C57BL/6j) were kept in the animal house and fed *ad libitum* and anesthetized by isoflurane inhalation before euthanasia.

### 2.2 Slice Preparation and Solutions

After brain removal, transversal (horizontal) hippocampal slices with a 350-μm thickness were prepared from 3- to 6-postnatal-week-old mice using a Leica VT1200 (Leica, Wetzlar, Germany) as recently described.^28^^,^^39^ The extracellular solution contained (in mM): 125 NaCl, 26 ${\mathrm{NaHCO}}_{3}$, 20 glucose, 3 KCl, 1 ${\mathrm{NaH}}_{2}{\mathrm{PO}}_{4}$, and 2 ${\mathrm{CaCl}}_{2}$ bubbled with 95% $O_{2}$ and 5% ${\mathrm{CO}}_{2}$. After slicing, slices were pre-incubated for 45 min at 37°C and then maintained at room temperature. During experiments, slices were perfused in the recording chamber with extracellular solution at 32°C to 34°C. In some experiments, the following chemicals were added to the extracellular solution: tetrodotoxin (TTX), 2,3-dioxo-6-nitro-1,2,3,4-tetrahydrobenzo[f]quinoxaline-7-sulfonamide disodium salt (NBQX), and d-(-)-2-amino-5-phosphonopentanoic acid (D-AP5). All chemicals were from Hello Bio (Dunshaughlin, Republic of Ireland), and they were pre-dissolved in water before being diluted to the final concentration indicated in Sec. 3.

### 2.3 Slice Loading with Ca^2+^ Indicators and Staining with EF-630 VSD

To load ${\mathrm{Ca}}^{2+}$ indicators, slices were incubated at room temperature for 30 to 45 min with an extracellular solution containing 1 to 2  μM of the AM-ester pre-dissolved in DMSO at 5 mM. Assessed ${\mathrm{Ca}}^{2+}$ indicators were Fluo-4, Fluo-8, Cal520, and Calbryte520 (AAT Bioquest, Pleasanton, California, United States). Staining with EF-630 (Potentiometric Probes, Farmington, Connecticut, United States) was done directly in the recording chamber with a procedure similar to that developed in another laboratory.^38^ In detail, continuous prefusion was stopped, and the volume of the extracellular solution was reduced to ∼200  μL. Three microliters of a solution containing 500-μM EF-630 dissolved in $H_{2}O$ was added, and the slice was kept for ∼1  min under ∼7.5-μM EF-630 before perfusion was restarted. In combined imaging experiments, Calbryte520 loading was done before EF-630 staining.

### 2.4 Optical Arrangement, Electrical Stimulation, and Imaging

**Fig. 1:**
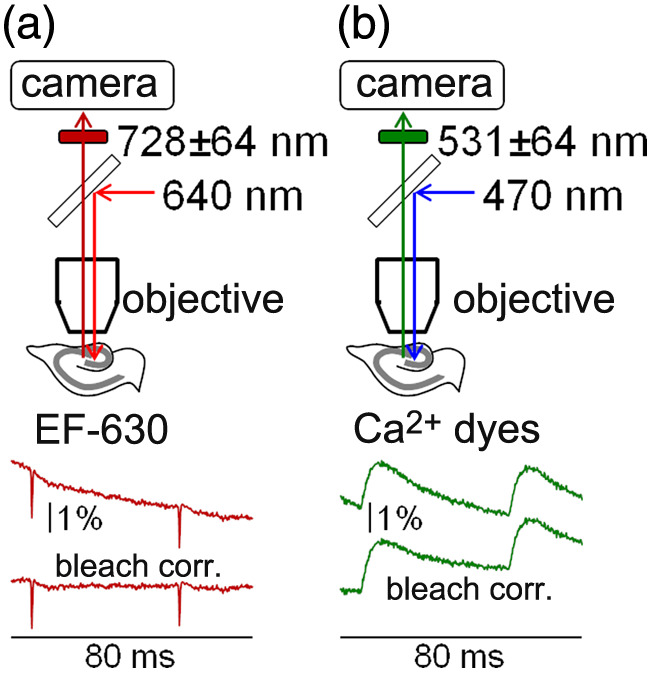
Scheme of the optical pathways used for the combination of two indicators and bleach correction. (a) Top: for the VSD EF-630, fluorescence was excited at 640 nm and emitted light band-pass filtered at 728±64  nm before camera acquisition. Bottom: raw EF-630 signal from a 100×100 ROI before and after bleach correction in a representative example of combined $V_{m}$ and ${\mathrm{Ca}}^{2+}$ imaging. (b) For ${\mathrm{Ca}}^{2+}$ indicators, fluorescence was excited at 470 nm and emitted light band-pass filtered at 530±21  nm before camera acquisition. Bottom: raw ${\mathrm{Ca}}^{2+}$ fluorescence signal from the same experiment and in the same 100×100 ROI before and after bleach correction.

The setup, based on an Olympus BX51 microscope equipped with a 25×/1.05 NA objective (model XLPLN25XWMP2), was used in recent studies.^28^^,^^39^ The diameter of the field of view of this objective is ∼720  μm. As previously described,^28^^,^^39^ to image with a pixel resolution of ∼1  μm from the largest possible portion of the hippocampus, images projected to a Kinetix (Teledyne Photometrics, Tucson, Arizona, United States) CMOS camera, used for imaging, were demagnified by 0.25×. In the mouse hippocampus, this arrangement allowed visualizing with ∼750  pixels in the X-direction the last part of the CA3 pyramidal cell layer and most of the CA1 pyramidal cell layer. Images with ∼250  pixels in the Y-direction were acquired at a 5-kHz frame rate at 8-bit depth. For electrical stimulation, pipettes of 2 to 4  μM diameter filled with extracellular solution were used. Precisely, the pipette was positioned above the stratum lucidum corresponding to the last part of the mossy fiber pathway, i.e., of the CA3 region. Electrical activity was elicited by constant current pulses of 30- to 60-μA amplitude and 100-μs duration. EF-630 fluorescence was excited using the 640-nm line of an LDI-7 laser (89 North, Williston, Vermont, United States), and emitted fluorescence was band-pass filtered at 728±64  nm [Fig 1(a)]. ${\mathrm{Ca}}^{2+}$ fluorescence (from all indicators) was excited by a 470-nm OPTOLED (Cairn Research, Faversham, United Kingdom), and emitted fluorescence was band-pass filtered at 530±21  nm [Fig 1(b)].

### 2.5 Data Recording and Analysis

Images were acquired with the open source software Micro-Manager, and data were analyzed either in MATLAB or in Python. Frame sequences, either from single trials or from averages of two or seven trials with identical responses, were corrected for photo-bleaching in both $V_{m}$ [Fig 1(a)] and ${\mathrm{Ca}}^{2+}$ [Fig 1(b)] recordings. Signals were expressed as absolute $\Delta F/F_{0}$: $+\Delta F/F_{0}$ for ${\mathrm{Ca}}^{2+}$ fluorescence or $-\Delta F/F_{0}$ for $V_{m}$ fluorescence because depolarization corresponds to a decrease in fluorescence under our experimental conditions. In the whole article, they were calculated from regions of interest (ROIs) of either 50×50 or 100×100  pixels. In one figure, signals were illustrated in colored scales after applying twice a 50×50 median filter, first to the raw frames and then to the absolute $\Delta F/F_{0}$ frames. Finally, for quantification of slow signals beyond the noise, a Savitsky–Golay smoothing filter was utilized.^40^

### 2.6 Statistical Analysis

In this technical report, paired t-tests were applied to assess the following hypotheses: (1) whether a rundown of fluorescence transients occurred, (2) whether parameters of signals differed at different sites in the same experiment, and (3) whether delivery of a chemical had an effect on signal parameters. The test was applied to N=5 slices when the effect of TTX was assessed, and to groups of N=7 slices in all other cases. Values of P below certain thresholds (either 0.01 or 0.001) are reported in the text to support significant differences.

## 3 Results

### 3.1 EF-630 Fluorescence Transients Following CA3 Electrical Stimulation

**Fig. 2:**
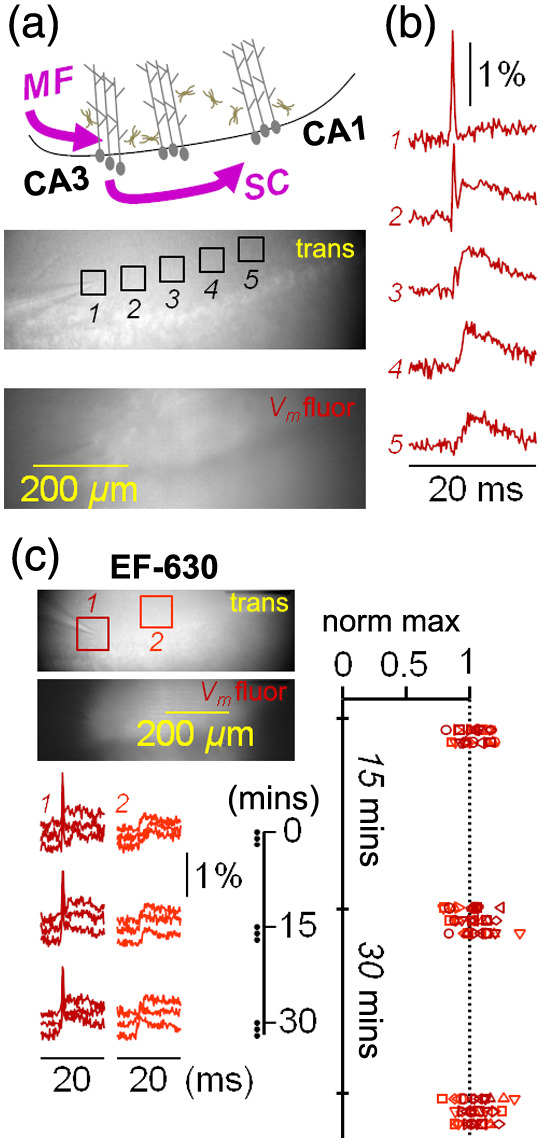
EF-630 transients in hippocampal slices. (a) Top: scheme of imaged hippocampal portions; the stimulation electrode is placed above the ending of the MF pathway; the recording area includes most of the CA1 region where Shaffer collaterals form synaptic contacts with pyramidal neurons. Bottom: transmitted light (trans) image and EF-630 fluorescence ($V_{m}$ fluor) image of the recording area (760×251  pixels) in a hippocampal slice; five ROIs of 50×50  pixels are indicated. (b) EF-630 ($V_{m}$) transients ($-\Delta F/F_{0}$), from ROIs in panel (a), associated with a stimulation pulse occurring at t∼8  ms in a 20-ms recording; a spike is detected in ROIs 1 and 2; depolarizations with EPSP shape are detected in ROIs 2 to 5. (c) Left-top: in another slice trans and $V_{m}$ fluor images of the recording area; two 100×100  pixel ROIs are indicated. Left-bottom: nine consecutive $V_{m}$ transients from the two ROIs above associated with a stimulation pulse; the first three recordings were initially performed every minute; the next three recordings were performed every minute after 15 min; the last three recordings were performed every minute 30 min after the first recording; spikes are detected in ROI 1 (dark red), and EPSPs are detected in ROI 2 (light red). Right: scatter plots of EF-630 transients maxima normalized to maxima of the first recording from N=7 slices where the test of the example illustrated on the left was performed.

As described in Sec. 2, transversal slices of the hippocampus were loaded with the VSD EF-630 by exposing them to the dye in the recording chamber. After that, a portion of the pyramidal cell layer comprising the final part of the CA3 region and the initial part of the CA1 region was visualized [scheme on the top of Fig. 2(a)]. As in experiments presented in an earlier report,^28^ a stimulating electrode was positioned above the stratum lucidum at the end of the mossy fiber (MF) pathway. A transmitted light image and an EF-630 fluorescence image after staining are shown in Fig. 2(a) (bottom). At the same spatial resolution, fluorescence images could be acquired at 5k frames/s. In this example, five ROIs of 50×50  pixels ($50\times 50\;{\mu m}^{2}$) were selected, the first one (ROI 1) centered on the tip of the stimulating electrodes and the others (ROIs 2 to 5) in the stratum radiatum at progressively longer distances toward the CA1 region. Fluorescence transients (averages of seven trials) from the five ROIs are shown in Fig. 2(b). At ROIs 1 and 2, a spike with a clear shape of an AP, presumably occurring in directly stimulated neuronal populations, was detected just after the stimulation pulse. At ROIs 2 to 5, smaller depolarization transients were observed. These signals have shape of EPSPs, but depolarization in these neuronal populations can be boosted by asynchronous firing elicited by the EPSPs. In agreement with what has been reported by another laboratory,^38^ we noticed that individual fluorescence transients were stable over time for many trials. As we estimate that 30 min is a reasonable time window necessary for many types of studies in brain slices, we quantitatively assessed the stability of EF-630 over this time window. In the example of Fig. 2(c), fluorescence transients in single trials, from a 100×100  pixel ROI (1) centered on the tip of the stimulator, and from another 100×100  pixel ROI (2) centered at ∼200  μm from the first one, are shown. We used larger ROIs in this case to quantify fluorescence transients from single trials. A stimulation pulse was delivered three times every minute, then three times after 15 min, and finally three times after 30 min from the first stimulation. Transients from both ROI 1 (dark red) and ROI 2 (light red) were stable over time with fluctuations within the noise. We performed this test in N=7 slices and obtained the same result [scatter plots in Fig. 2(c)]. We concluded that EF-630 imaging can reliably monitor neuronal excitability and synaptic transmission in healthy brain slices for at least 30 min. As the aim of this study was to characterize the ability of combining EF-630 imaging with ${\mathrm{Ca}}^{2+}$ imaging using blue-excitable green-emitting indicators, we assessed ${\mathrm{Ca}}^{2+}$ indicators in the same manner.

### 3.2 Assessment of Four Blue-Excitable Green-Emitting Ca^2+^ Indicators

**Fig. 3:**
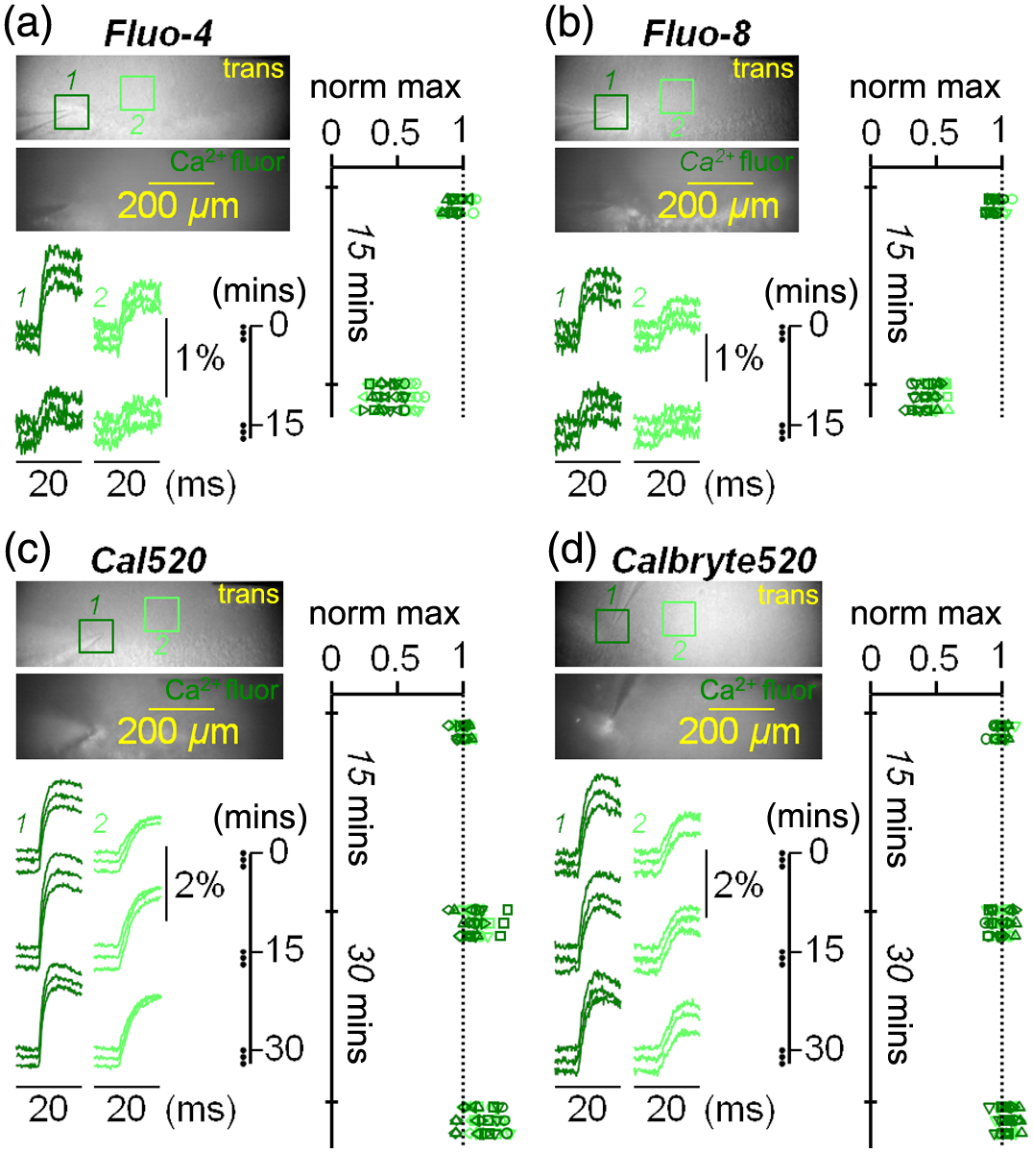
${\mathrm{Ca}}^{2+}$ transients from four different indicators in hippocampal slices. (a) Left-top: in a slice loaded with Fluo-4, trans and ${\mathrm{Ca}}^{2+}$ fluor images of the recording area; two 100×100  pixel ROIs are indicated. Left-bottom: six consecutive ${\mathrm{Ca}}^{2+}$ transients from the two ROIs above associated with a stimulation pulse; the first three recordings were initially performed every minute; the next three recordings were performed every minute after 15 min. Right: scatter plots of EF-630 transients maxima normalized to maxima of the first recording from N=7 slices where the test of the example illustrated on the left was performed; a rundown of signals after 15 min is observed. (b) Same as panel (a) but for slices loaded with Fluo-8. (c) Same as panels (a) and (b) for slices loaded with Cal520 but, in this case, with three additional recordings 30 min after the first recording; no rundown is observed in scatter plots on the right. (d) Same as panel (c) for slices loaded with Calbryte520; also with this indicator, no rundown is observed in scatter plots.

We recently established the ability of the ${\mathrm{Ca}}^{2+}$ indicator Fluo-4, previously used to monitor large slow signals from glial cells^26^^,^^27^ to report neuronal activity.^28^ Using this dye, however, we observed a rundown of $\Delta F/F_{0}$ signals occurring within a few minutes. In the example of Fig. 3(a), a pulse of stimulation was initially given three times every minute. Fluorescence transients were shown from a 100×100  pixel ROI, centered on the tip of the stimulator (dark green), and from another 100×100  pixel ROI, centered at ∼200  μm from the first one (light green). The first three signals recorded every minute were stable in both regions, but when we performed the next three recordings after 15 min, signals were appreciably smaller. The same behavior was observed in N=7 slices where Fluo-4 was tested [see scatter plots on Fig. 3(a)], and the mean of the three signals recorded 15 min after the first trial was significantly smaller with respect to the first signal (P<0.001, paired t-test). As Fluo-4 cannot perform over time as EF-630, we next assessed the similar indicator Fluo-8. As shown in the example of Fig. 3(b) and in the scatter plots on the right, reporting the measurement in N=7 slices, Fluo-8 signals are affected by rundown similar to that observed in slices loaded with Fluo-4. Also, for Fluo-8, signals recorded 15 min after the first trial were significantly smaller than the first signal (P<0.001, paired t-test). In a comparative study where several ${\mathrm{Ca}}^{2+}$ indicators were tested,^41^ it was established that Cal520^42^ performs better than Fluo-4 and Fluo-8, and we therefore assessed this dye. As shown in the example of Fig. 3(c), Cal520 gives transients that are larger than those from the previous two indicators, but more importantly, these transients did not exhibit rundown when the three recordings were repeated first after 15 min and then after 30 min. However, as shown in the scatter plots from N=7 slices on the right Fig. 3(c), Cal520 transients tend to increase in size over time, on average by ∼8% every 5 min. We therefore assessed another ${\mathrm{Ca}}^{2+}$ indicator (Calbryte520) that was reported to have larger retention with respect to Fluo-4.^43^ Calbryte520 gave transients that are similar in size to those of Cal520 and also these signals had no rundown when recordings were repeated over 30 min, a behavior observed in all N=7 slices assessed with this dye [scatter plots on Fig. 3(d)]. However, in contrast to Cal520, no tendency to increase for signals with Calbryte520 was observed over time. We concluded that both Cal520 and Calbryte520 do not exhibit rundown over time, but that Calbryte520 is preferable to be combined with EF-630 for stable recordings over at least 30 min.

### 3.3 Combining EF-630 and Calbryte520 Fluorescence Recordings

**Fig. 4:**
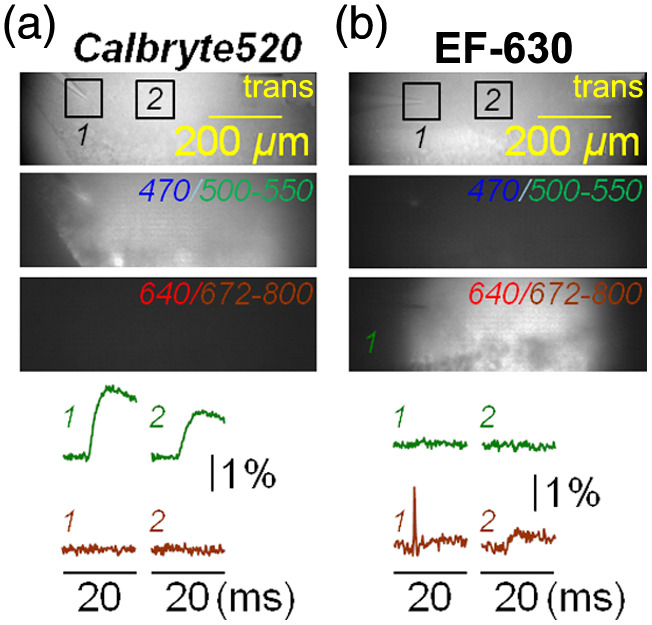
Absence of crosstalk between EF-630 and Calbryte520. (a) Top: in a slice loaded with Calbryte520 only, trans image and images obtained with epi-fluorescence excitation at 470 nm and emission band-pass filtered at 531±21  nm or with epi-fluorescence excitation at 640 nm and emission band-pass filtered at 728±64  nm. Bottom: fluorescence transients from ROIs 1 and 2 illustrated above, using the two epi-fluorescence spectral windows; ${\mathrm{Ca}}^{2+}$ transients were observed only in the green-emitting window. (b) Same as panel (a) but in a slice with EF-630 only. $V_{m}$ transients were observed only in the red IR-emitting window. The lookup scale of fluorescence images is the same, whereas the 640-nm laser power was >3 times higher in the image of panel (a) and the 470-nm LED power >3 times higher in the image of panel (b).

**Fig. 5:**
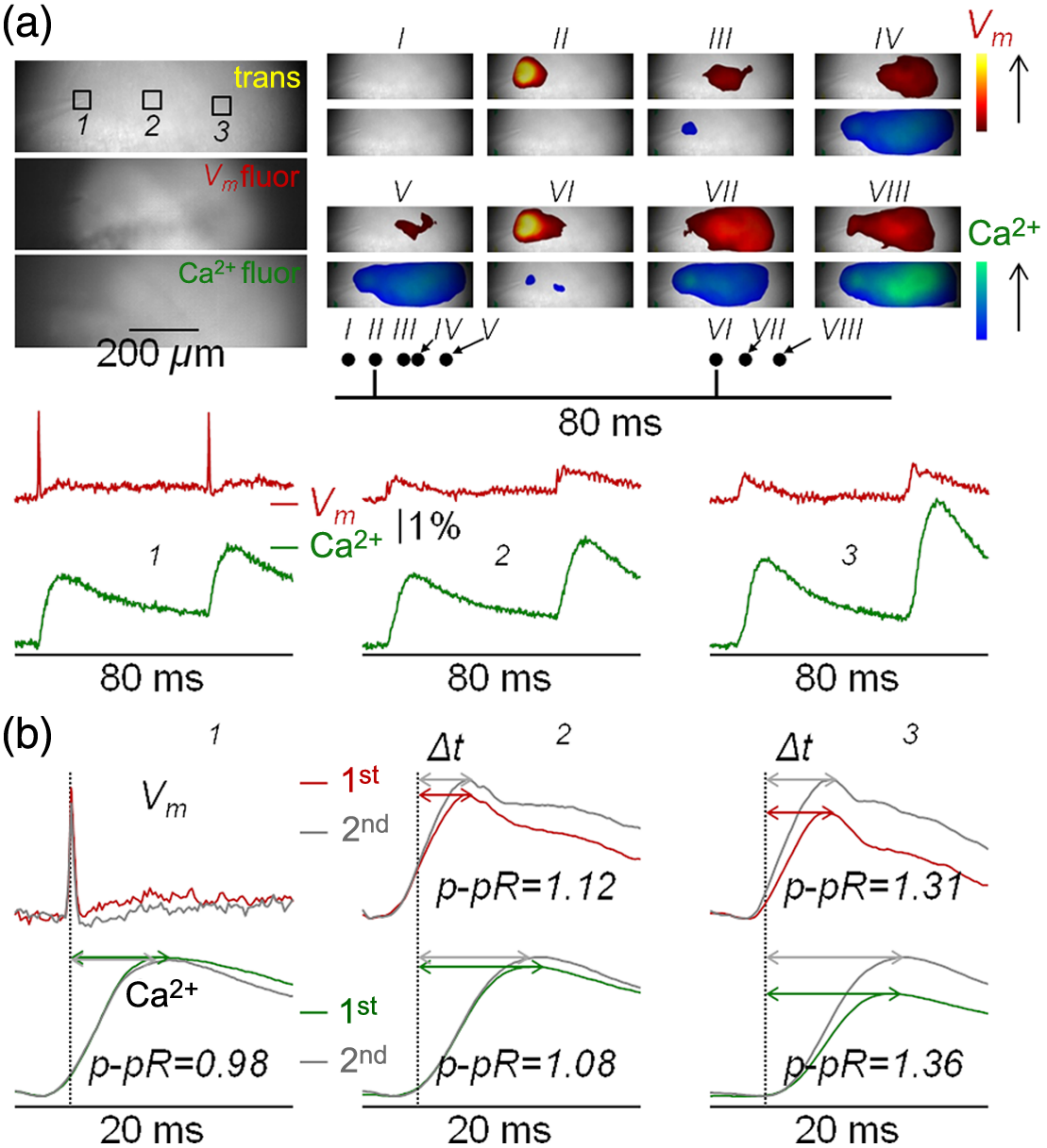
Combined EF-630 and Calbryte520 imaging. (a) Top-left: trans, $V_{m}$ fluor, and ${\mathrm{Ca}}^{2+}$ fluor images of the recording area with three ROIs of 50×50  pixels indicated. Top-right: spatial evolution of $V_{m}$ and ${\mathrm{Ca}}^{2+}$ transients elicited by paired-pulse stimulation captured from eight instants (I to VIII) illustrated using two color scales on the right. Bottom: $V_{m}$ (average of seven trials) and ${\mathrm{Ca}}^{2+}$ (average of two trials) transients from the three ROIs; a spike is detected in ROI 1, and EPSPs are detected in ROIs 2 and 3. (b) Top-left: traces are spikes in ROI 1 associated with the first and second stimuli of the paired-protocols superimposed. The other traces are the $V_{m}$ and ${\mathrm{Ca}}^{2+}$ transients in the ROIs indicated smoothed by a Savisky–Golay filter. The p-pR of EPSPs and ${\mathrm{Ca}}^{2+}$ transients are reported, and delays between the spike and signal maxima (Δt) are indicated by double-headed arrows.

Combining optimal measurements from two independent indicators requires no crosstalk between the two excitation/emission channels, i.e., fluorescence signals from each indicator must be negligible when excited and recorded at the wavelengths of the other indicator. Calbryte520 has fluorescence spectra similar to fluorescein: fluorescence is not excited by red light, and there is no IR emission. Consistently, in the slice of Fig. 4(a) loaded with Calbryte520 only, ${\mathrm{Ca}}^{2+}$ transients were measured when fluorescence was excited at 470 nm and recorded at 530±21  nm, but not when it was excited at 640 nm and recorded at 728±64  nm, even using >3 times higher laser power of what was used for EF-630 imaging. In the case of EF-630, the shortest wavelengths of the excitation spectrum include blue wavelengths, but the VSD does not emit green light. Thus, in the slice of Fig. 4(b) loaded with EF-630 only, $V_{m}$ transients were measured when fluorescence was excited at 640 nm and recorded at 728±64  nm, but not when it was excited at 470 nm and recorded at 530±21  nm, even using >3 times the LED power of what was used for Calbryte520 imaging. Although the possibility of simultaneous EF-630 and Calbryte520 recordings would require tests with dual-wavelength excitation, the present tests demonstrate that sequential recordings can be reliably achieved to obtain independent $V_{m}$ and ${\mathrm{Ca}}^{2+}$ measurements. In the example of Fig. 5(a) (average of seven trials for $V_{m}$ recordings and two trials for ${\mathrm{Ca}}^{2+}$ recordings), we delivered two stimuli at 50-ms interval. The spatial evolution of $V_{m}$ and ${\mathrm{Ca}}^{2+}$ optical transients, captured from eight instants (I to VIII) is illustrated using two distinct color scales on the right. In the area adjacent to the electrode, the “spike” associated with direct stimulation precedes the ${\mathrm{Ca}}^{2+}$ transient, and it also precedes “EPSPs” in the CA1 region. To analyze the signals, we focused in this case on three ROIs of 50×50  pixels: ROI 1, centered on the tip of the stimulator; ROI 2, centered at ∼200  μm from the stimulator; and ROI 3 centered at ∼400  μm from the stimulator [Fig. 5(a), bottom traces]. Interestingly, at ROIs 2 and 3, both $V_{m}$ and ${\mathrm{Ca}}^{2+}$ signals associated with the second pulse were larger with respect to the signals associated with the first pulse, a result which is consistent with the known phenomenon of paired-pulse facilitation.^44^^–^^46^ To quantitatively assess signals beyond the noise, ${\mathrm{Ca}}^{2+}$ traces in ROI 1 and all traces in ROIs 2 and 3 were smoothed with a Savitsky–Golay algorithm. Signals on time windows of 20 ms with the first and second spikes occurring after 8 ms are shown in Fig. 5(b). The paired-pulse ratios (p-pRs) of EPSPs and ${\mathrm{Ca}}^{2+}$ transients and the delays between the spike and signal maxima (Δt) were calculated, in this way providing for each slice experiment a set of 15 quantitative parameters.

Values of these parameters obtained in N=12 slices are reported in Table S1 in the Supplementary Material. In the directly stimulated ROI 1, ${\mathrm{Ca}}^{2+}$ p-pR was 1.0±0.06, and Δt values were 6.7±0.6 and 6.4±0.7  ms for the first and second pulse, respectively, suggesting that these signals are produced by ${\mathrm{Ca}}^{2+}$ influx in directly stimulated cells. $V_{m}$ p-pR was 1.41±0.32 in ROI 2 and 1.62±0.36 in ROI 3, but ${\mathrm{Ca}}^{2+}$ p-pR was only 1.11±0.16 in ROI 2 and 1.22±0.19 in ROI 3. The qualitative observation of smaller p-pR values for ${\mathrm{Ca}}^{2+}$ with respect to $V_{m}$ suggests that an important fraction of the ${\mathrm{Ca}}^{2+}$ transient does not have a postsynaptic origin. In ROI 2, at ∼200  μm from the electrode, $V_{m}$ Δt values were 4.1±0.7 and 4.3±0.6  ms for the first and second pulse, respectively. These values were significantly shorter (P<0.01, paired t-test) than those in ROI 3 at ∼400  μm from the electrode: 4.9±0.7 and 5.1±0.5  ms. Finally, in ROI 2, ${\mathrm{Ca}}^{2+}\Delta t$ values were 8.4±1.0 and 8.7±1.3  ms for the first and second pulse, respectively. In ROI 3, ${\mathrm{Ca}}^{2+}$ Δt values were 10.0±1.0  ms and 9.6±1.3  ms, for the first and second pulse, respectively. Finally, in both ROIs 2 and 3 and for both first and second pulses, $V_{m}$ Δt was significantly shorter than ${\mathrm{Ca}}^{2+}$ Δt (P<0.001, paired t-test). Quantitative parameters introduced in this section were used to characterize the origin of EF-630/Calbryte520 signals.

### 3.4 Initial Characterization of the Origin of EF-630/Calbryte520 Signals

**Fig. 6:**
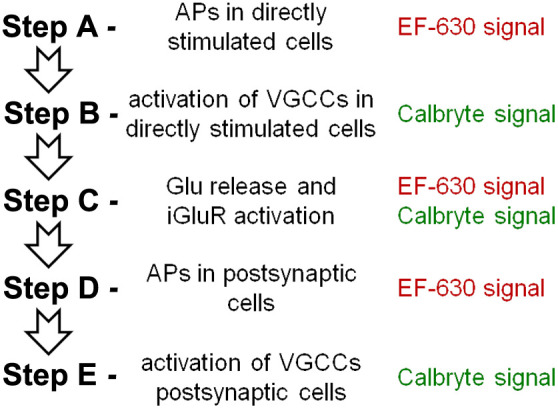
Expected sequential steps after stimulation. (Step A) APs in a cell population are elicited directly by the stimulation producing EF-630 signals. (Step B) Propagating APs activate VGCCs producing Calbryte520 signals. (Step C) Glutamate release activates iGluRs generating EPSPs that produce both EF-630 and Calbryte520 signals. (Step D) EPSPs can generate postsynaptic APs producing EF-630 signals. (Step E) Postsynaptic APs activate VGCCs producing Calbryte520 signals.

**Fig. 7:**
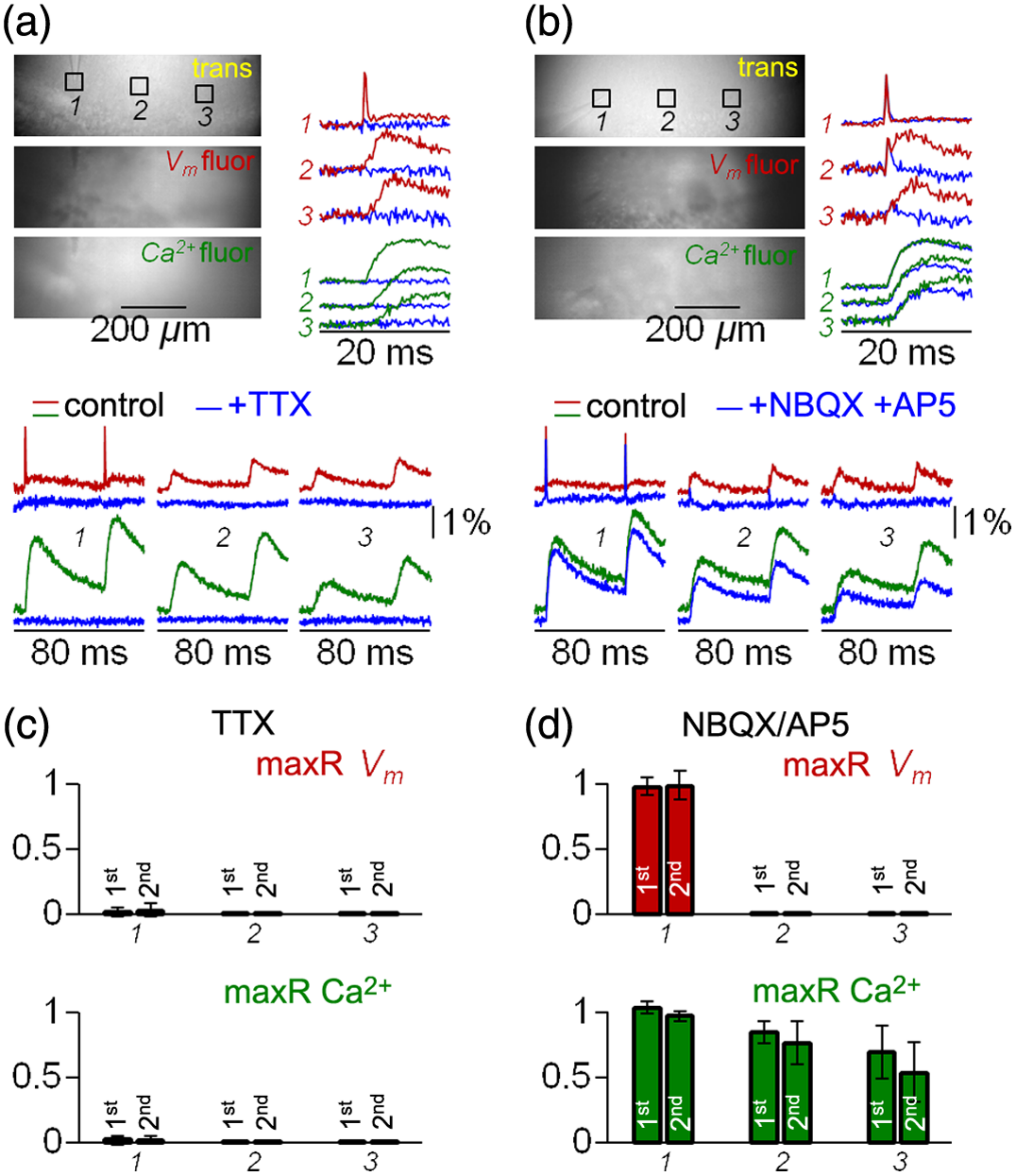
Effects of TTX and iGluR inhibitors on $V_{m}$ and ${\mathrm{Ca}}^{2+}$ transients. (a) Top-left: trans, $V_{m}$ fluor and ${\mathrm{Ca}}^{2+}$ fluor images of the recording area with three ROIs of 50×50  pixels indicated (the same relative position as in Fig. 5). Bottom: average $V_{m}$ (seven trials) and ${\mathrm{Ca}}^{2+}$ (two trials) transients elicited by paired-pulse stimulation from the three ROIs under control conditions and after delivery of 1  μM TTX (blue traces). Top-right: $V_{m}$ and ${\mathrm{Ca}}^{2+}$ averaged transients associated with the first stimulation pulse, in control and after TTX delivery, are superimposed. (b) Same as panel (a) but in an example of delivery of 10  μM NBQX and 50  μM D-AP5 (iGluR inhibitors). (c) Mean ± SD (N=5 slices) of the ratios between signal maxima (maxR) after and before TTX delivery: top, $V_{m}$ transients; bottom, ${\mathrm{Ca}}^{2+}$ transients. (d) Same as in panel (c) but for N=7 slices where delivery of iGluR inhibitors was tested.

**Fig. 8:**
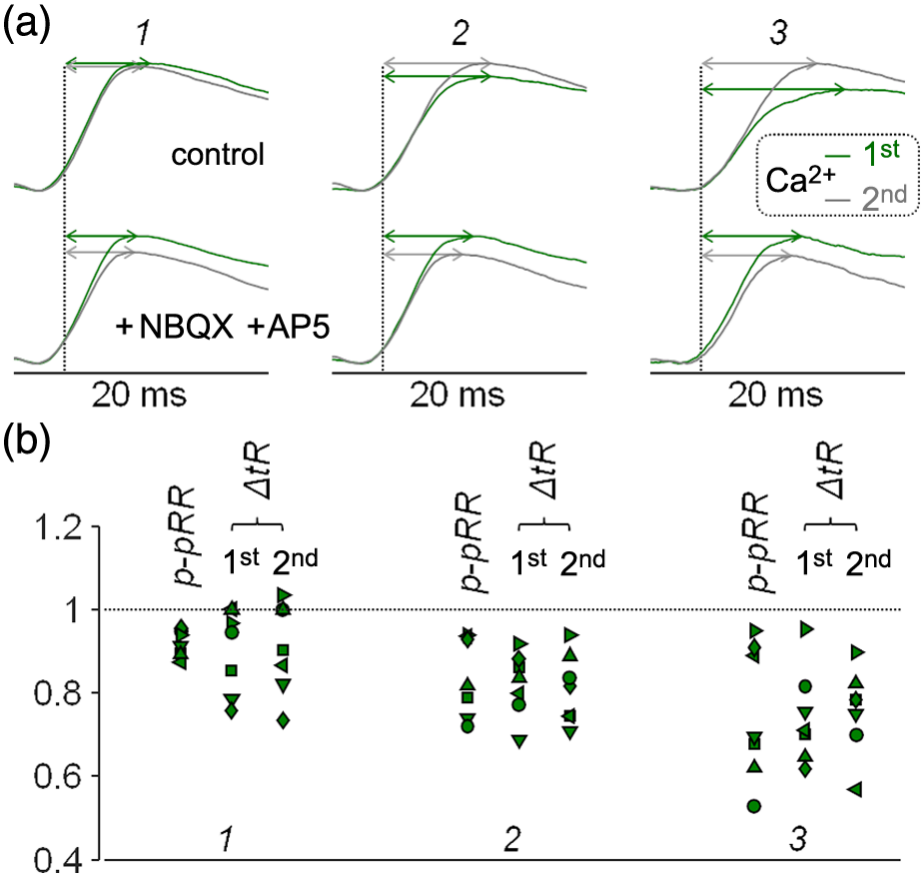
Effects iGluR inhibitors on ${\mathrm{Ca}}^{2+}$ p-pR and Δt parameters. (a) ${\mathrm{Ca}}^{2+}$ transients of Fig. 7(b) in the three ROIs indicated smoothed by a Savisky–Golay filter; the transients associated with the first and second stimulating pulses are superimposed; delays between the spike and signal maxima (Δt) are indicated by double-headed arrows; top traces are in control conditions; bottom traces are after delivery of iGluR inhibitors. (b) Scatter plots in the three ROIs of the ratios between p-pR (p-pRR) and Δt (ΔtR) parameters after delivery of NBQX and D-AP5 and in control conditions from N=7 slices where iGluR inhibitors were tested.

The final goal of this pilot study was to link experimental results to the electrical and ${\mathrm{Ca}}^{2+}$ activity of the slice. The scheme of Fig. 6 illustrates the sequential steps expected after stimulation. First (step A), APs in a cell population are elicited directly by stimulation, producing EF-630 signals. These propagating APs activate voltage-gated ${\mathrm{Ca}}^{2+}$ channels (VGCCs, step B) producing Calbryte520 signals and neurotransmitter release. If the neurotransmitter is glutamate, this activates ionotropic glutamate receptors (iGluRs, step C) and EPSPs that can be measured with EF-630, but ${\mathrm{Ca}}^{2+}$ influx via iGluRs can also produce Calbryte520 signals. Postsynaptic depolarization above firing thresholds can boost EF-630 signals (step D), activating postsynaptic VGCCs that generate Calbryte signals (step E). The scheme can be repeated in further steps in case of polysynaptic activity. As these steps are sequential, selective intervention at each step would modify the following steps leaving the previous ones unaltered. Thus, to provide an initial characterization of EF-630/Calbryte520 signals, we blocked step A (presynaptic APs) by delivering 1  μM of the ${\mathrm{Na}}^{+}$ channel inhibitor TTX, or we blocked step C (iGluRs activation) by delivering a cocktail of the AMPA receptors inhibitor NBQX (10  μM) and of the NMDA receptors inhibitor D-AP5 (50  μM). As shown in the representative example of Fig. 7(a), TTX delivery blocks $V_{m}$ and ${\mathrm{Ca}}^{2+}$ signals in all ROIs (described in Sec. 3.3), as expected from the scheme. In contrast, as shown in the representative example of Fig. 7(b), iGluRs inhibition blocks EPSPs at ROIs 2 to 3 but has negligible effect on APs and ${\mathrm{Ca}}^{2+}$ transients at ROI 1, as expected from the fact that these signals are produced at steps A and B. Notably, Calbryte520 transients at ROIs 2 to 3 were reduced but not fully blocked by NBQX and D-AP5 delivery, indicating that residual signals originate at steps A and B and are therefore from presynaptic axons of directly stimulated neurons. The recording of $V_{m}$ and ${\mathrm{Ca}}^{2+}$ signals following the paired-pulse protocol before and after TTX delivery was performed in N=5 slices. Transients were blocked in all slices (P<0.001, paired t-test), and bar diagrams of Fig. 7(c) show the mean ± SD of the ratios between signal maxima (maxR) after and before TTX delivery. The recording of $V_{m}$ and ${\mathrm{Ca}}^{2+}$ signals following the paired-pulse protocol before and after delivery of iGluR inhibitors was performed in N=7 slices, and bar diagrams of the mean ± SD of maxR are shown in Fig. 7(d). In all slices, APs and ${\mathrm{Ca}}^{2+}$ transients in ROI 1 were unaffected, whereas EPSPs in ROIs 2 to 3 were blocked (P<0.001, paired t-test). Yet, ${\mathrm{Ca}}^{2+}$ transients at ROIs 2 to 3 were reduced but not fully blocked by delivery of iGluR inhibitors. Experiments of Figs. 7(b) and 7(d) were used to evaluate the quantitative parameters related to ${\mathrm{Ca}}^{2+}$ transients reported in Table S1 in the Supplementary Material. ${\mathrm{Ca}}^{2+}$ transients of Fig. 7(b), smoothed with a Savitsky–Golay filter, are shown in Fig. 8(a). Specifically, as ${\mathrm{Ca}}^{2+}$ transients associated with the first and second stimulation pulse were superimposed, it was evident in ROIs 2 and 3, but less in ROI 1, that delivery of iGluR inhibitors decreased the p-pR and the delay between spike and ${\mathrm{Ca}}^{2+}$ signal peaks (Δt). Scatter plots in Fig. 8(b) show the ratios of p-pR (p-pRR) and of Δt (ΔtR) parameters in the seven slices reported in Fig. 7(d). Delivery of NBQX and D-AP5 systematically reduced p-pR and Δt of ${\mathrm{Ca}}^{2+}$ transients in both ROIs 2 and 3 (P<0.01 paired t-test). The first result indicates that ${\mathrm{Ca}}^{2+}$ paired-pulse facilitation in control conditions is mediated by glutamatergic synaptic transmission. The second result gives a quantitative estimate between the presynaptic and postsynaptic components of ${\mathrm{Ca}}^{2+}$ transients.

## 4 Discussion

In the present article, we report substantial improvements in brain slice $V_{m}$ and ${\mathrm{Ca}}^{2+}$ imaging achieved using the combination of the VSD EF-630 and the ${\mathrm{Ca}}^{2+}$ indicator Calbryte520. The brain slice is an *ex vivo* preparation that allows investigation of neurons in their physiological environment, where many connections that form local networks are preserved. At present, it is possible to investigate brain slice activity in specific cell types using genetically encoded fluorescence indicators both for $V_{m}$ (GEVI)^47^^–^^49^ and ${\mathrm{Ca}}^{2+}$ (GECI).^50^^,^^51^ These state-of-the-art approaches achieve relatively large (>1%) fluorescence transients, but they encounter several technical challenges starting from the need of expressing the protein in a living animal but also caveats related to the kinetics of indicators. For example, a remarkable temporal discrepancy between GEVI and GECI imaging was reported in brain slices.^52^ Organic indicators, in contrast, do not offer the possibility to target cell classes with specific molecular identity, but the possibility to record fluorescence transients with a size comparable to those from GEVIs or GECIs can make their use the preferable choice in many instances. Slices can be prepared in standard ways from any animal model and only later loaded with the ${\mathrm{Ca}}^{2+}$ indicator, and rapidly stained with the VSD just before recording. State-of-the-art CMOS cameras can be used to record fluorescence at high spatiotemporal resolution with stable signals lasting for >30  min. In addition, the large gap between fluorescence spectra of EF-630 and Calbryte520 allows recordings of fully independent $V_{m}$ and ${\mathrm{Ca}}^{2+}$ signals associated with the same neuronal activity. Notably, the combination of blue/red excitation still leaves the possibility of a third independent illumination pathway in the UV range that can be used, for instance, for uncaging techniques.^53^ Alternatively, by giving up ${\mathrm{Ca}}^{2+}$ imaging, blue illumination can be used for channelrhodopsin excitation in combination with $V_{m}$ imaging.^54^ The spectral properties of EF-630, as well as its large sensitivity, also allow combining this dye with GFP-based genetically encoded indicators for ${\mathrm{Ca}}^{2+}$, glutamate, or voltage.^55^ Specifically, combining a GEVI with an organic VSD can provide a more comprehensive understanding of the circuit under investigation because a GEVI typically labels only one cell type and sometimes only a specific cellular compartment.

The outstanding SNR of the measurements obtained at very high spatiotemporal resolution that are reported in this article allowed an initial characterization of fluorescence transients elicited by electrical stimulation of the hippocampal CA3 region. Clear APs from the population of directly stimulated hippocampal neurons are distinguishable, as well as monosynaptic EPSPs in the CA1 region where Shaffer collaterals are expected to make connections. These EPSPs were fully blocked by delivery of iGluR inhibitors [Fig. 6(b) and 6(d)]. A straightforward correlation between $V_{m}$ and ${\mathrm{Ca}}^{2+}$ signals, however, is not possible because ${\mathrm{Ca}}^{2+}$ signals at the EPSP sites comprise a combination of postsynaptic and presynaptic ${\mathrm{Ca}}^{2+}$ influx. This result is not surprising because EF-630 staining and Calbryte520 loading are independent and therefore fluorescence transients do not, in general, originate from the same cells (or, if they do, not in the same proportion). Nevertheless, the accurate analysis of signal kinetics permitted by the high temporal resolution (5 kHz) allowed identifying different components of the ${\mathrm{Ca}}^{2+}$ transients and elucidating the observation of paired-pulse facilitation in postsynaptic cells. Full characterization will require more pharmacological analysis that may include delivery of D-AP5 alone to reveal ${\mathrm{Ca}}^{2+}$ influx via NMDA receptors, blockade of VGCCs, or blockade of $K^{+}$ channels that may result in the AP waveform changes. Such pharmacological investigations, however, are beyond the scope of the present technical article aimed at reporting a methodological step forward in combined $V_{m}$ and ${\mathrm{Ca}}^{2+}$ imaging *ex vivo*.

## 5 Conclusions

Achievements reported in this article open the door to a variety of potential studies in different brain regions (hippocampus, neocortex, thalamus, cerebellum, spinal cord, etc.), as well as in animal models of disease (epilepsy, autism, ataxia, migraine, etc.). They also lift previous limitations in detailed drug investigations of $V_{m}-{\mathrm{Ca}}^{2+}$ signaling.

## Supplementary Material

10.1117/1.NPh.13.S2.S23206.s01

## Data Availability

Frame sequences that were quantitatively analyzed, either single trial of averages of 2 or 7 trials as indicated in the text, are available in the public repository Zenodo (doi: 10.5281/zenodo.17725316). For data analysis, standard MATLAB or Python functions were used, and MATLAB scripts were written only to fasten data handling. These scripts are available upon request from the corresponding author.

## References

[1] Liang J. et al., “Monitoring population membrane potential signals from neocortex,” Adv. Exp. Med. Biol. 859, 171–196 (2015). AEMBAP 0065-2598 10.1007/978-3-319-17641-3_7 26238053

[2] Wright B. J. Jackson M. B., “Voltage imaging in the study of hippocampal circuit function and plasticity,” Adv. Exp. Med. Biol. 859, 197–211 (2015). AEMBAP 0065-2598 10.1007/978-3-319-17641-3_8 26238054

[3] Smetters D. Majewska A. Yuste R., “Detecting action potentials in neuronal populations with calcium imaging,” Methods 18, 215–221 (1999). MTHDE9 1046-2023 10.1006/meth.1999.0774 10356353

[4] Kékesi O. Keembiyage N. Buskila Y., “calcium imaging in brain tissue slices,” Methods Mol. Biol. 2861, 89–96 (2025). 10.1007/978-1-0716-4164-4_7 39395099

[5] Sugitani M. et al., “Optical imaging of the in vitro guinea pig piriform cortex activity using a voltage-sensitive dye,” Neurosci. Lett. 165, 215–218 (1994). NELED5 0304-3940 10.1016/0304-3940(94)90748-X 7912419

[r6] Nelson D. A. Katz L. C., “Emergence of functional circuits in ferret visual cortex visualized by optical imaging,” Neuron 15, 23–34 (1995). NERNET 0896-6273 10.1016/0896-6273(95)90061-6 7619527

[r7] Yuste R. Tank D. W. Kleinfeld D., “Functional study of the rat cortical microcircuitry with voltage-sensitive dye imaging of neocortical slices,” Cereb. Cortex 7, 546–558 (1997). 53OPAV 1047-3211 10.1093/cercor/7.6.546 9276179

[r8] Demir R. Haberly L. B. Jackson M. B., “Voltage imaging of epileptiform activity in slices from rat piriform cortex: onset and propagation,” J. Neurophysiol. 80, 2727–2742 (1998). JONEA4 0022-3077 10.1152/jn.1998.80.5.2727 9819277

[r9] Wu J. Y. Guan L. Tsau Y., “Propagating activation during oscillations and evoked responses in neocortical slices,” J. Neurosci. 19, 5005–5015 (1999). JNRSDS 0270-6474 10.1523/JNEUROSCI.19-12-05005.1999 10366633 PMC6782683

[r10] Laaris N. Carlson G. C. Keller A., “Thalamic-evoked synaptic interactions in barrel cortex revealed by optical imaging,” J. Neurosci. 20, 1529–1537 (2000). JNRSDS 0270-6474 10.1523/JNEUROSCI.20-04-01529.2000 10662842 PMC6772365

[11] Wu J. Y. et al., “Spatiotemporal properties of an evoked population activity in rat sensory cortical slices,” J. Neurophysiol. 86, 2461–2474 (2001). JONEA4 0022-3077 10.1152/jn.2001.86.5.2461 11698535

[12] Albowitz B. Kuhnt U., “Spatio-temporal distribution of epileptiform potentials in the hippocampal slice: recordings with voltage-sensitive dyes,” Eur. J. Neurosci. 3, 570–586 (1991). EJONEI 0953-816X 10.1111/j.1460-9568.1991.tb00844.x 12106489

[r13] Sinha S. R. Saggau P. J., “Imaging of 4-AP-induced, GABA(A)-dependent spontaneous synchronized activity mediated by the hippocampal interneuron network,” J. Neurophysiol. 86, 381–391 (2001). JONEA4 0022-3077 10.1152/jn.2001.86.1.381 11431518

[r14] Chang P.Y. Jackson M. B., “Heterogeneous spatial patterns of long-term potentiation in rat hippocampal slices,” J. Physiol. 576, 427–443 (2006). JPHYA7 0022-3751 10.1113/jphysiol.2006.112128 16873414 PMC1890346

[r15] Wright B. J. Jackson M. B., “Long-term potentiation in hilar circuitry modulates gating by the dentate gyrus,” J. Neurosci. 34, 9743–9753 (2014). JNRSDS 0270-6474 10.1523/JNEUROSCI.0814-14.2014 25031412 PMC4099549

[16] Jackson M. B., “Hebbian and non-Hebbian timing-dependent plasticity in the hippocampal CA3 region,” Hippocampus 30(12), 1241–1256 (2020). 10.1002/hipo.23252 32818312 PMC8565363

[17] Senseman D. M., “High-speed optical imaging of afferent flow through rat olfactory bulb slices: voltage-sensitive dye signals reveal periglomerular cell activity,” J. Neurosci. 16, 313–324 (1996). JNRSDS 0270-6474 10.1523/JNEUROSCI.16-01-00313.1996 8613798 PMC6578718

[18] Cohen D. Yarom Y., “Optical measurements of synchronized activity in isolated mammalian cerebellum,” Neuroscience 94, 859–866 (1999). 10.1016/S0306-4522(99)00348-6 10579576

[19] Chang P. Y. Jackson M. B., “Interpretation and optimization of absorbance and fluorescence signals from voltage-sensitive dyes,” J. Membr. Biol. 196, 105–116 (2003). JMBBBO 0022-2631 10.1007/s00232-003-0629-8 14724747

[20] Gusain P. et al., “Functional dissection of ipsilateral and contralateral neural activity propagation using voltage-sensitive dye imaging in mouse prefrontal cortex,” eNeuro 10, ENEURO.0161-23.2023 (2023). 10.1523/ENEURO.0161-23.2023 PMC10706255 37977827

[21] Utsumi Y. et al., “Assessing seizure liability in vitro with voltage-sensitive dye imaging in mouse hippocampal slices,” Front. Cell. Neurosci. 17, 1217368 (2023). 10.3389/fncel.2023.1217368 37680865 PMC10481167

[22] Albowitz B. König P. Kuhnt U., “Spatiotemporal distribution of intracellular calcium transients during epileptiform activity in guinea pig hippocampal slices,” J. Neurophysiol. 77, 491–501 (1997). JONEA4 0022-3077 10.1152/jn.1997.77.1.491 9120590

[23] Murayama M. et al., “Optical monitoring of progressive synchronization in dentate granule cells during population burst activities,” Eur. J. Neurosci. 21, 3349–3360 (2005). EJONEI 0953-816X 10.1111/j.1460-9568.2005.04167.x 16026472

[24] Mammano F. et al., “An optical recording system based on a fast CCD sensor for biological imaging,” Cell Calc. 25(2), 115–123 (1999). CECADV 0143-4160 10.1054/ceca.1998.0013 10326678

[25] Canepari M. et al., “GABA- and glutamate-mediated network activity in the hippocampus of neonatal and juvenile rats revealed by fast calcium imaging,” Cell Calc. 27, 25–33 (2000). CECADV 0143-4160 10.1054/ceca.1999.0086 10726208

[26] Parri H. R. Gould T. M. Crunelli V., “Spontaneous astrocytic Ca2+ oscillations in situ drive NMDAR-mediated neuronal excitation,” Nat. Neurosci. 4, 803–812 (2001). NANEFN 1097-6256 10.1038/90507 11477426

[r27] Parri H. R. Crunelli V., “The role of Ca2+ in the generation of spontaneous astrocytic Ca2+ oscillations,” Neuroscience 120, 979–992 (2003). 10.1016/S0306-4522(03)00379-8 12927204

[28] İpek Ö. Y. et al., “Fast neuronal calcium signals in brain slices loaded with Fluo-4 AM Ester,” Eur. J. Neurosci. 61, e16657 (2025). EJONEI 0953-816X 10.1111/ejn.16657 39804104 PMC11727817

[29] Sinha S. R. Patel S. S. Saggau P., “Simultaneous optical recording of evoked and spontaneous transients of membrane potential and intracellular calcium concentration with high spatio-temporal resolution,” J. Neurosci. Methods 60, 49–60 (1995). JNMEDT 0165-0270 10.1016/0165-0270(94)00219-7 8544487

[30] Sinha S. R. Saggau P., “Simultaneous optical recording of membrane potential and intracellular calcium from brain slices,” Methods 18, 204–214 (1999). MTHDE9 1046-2023 10.1006/meth.1999.0773 10356352

[31] Bullen A. Saggau P., “Indicators and optical configuration for simultaneous high-resolution recording of membrane potential and intracellular calcium using laser scanning microscopy,” Pflugers Arch. 436, 788–796 (1998). 10.1007/s004240050703 9716714

[32] Jaafari N. Marret E. Canepari M., “ Using simultaneous voltage and calcium imaging to study fast Ca(2+) channels,” Neurophotonics 2, 021010 (2015). 10.1117/1.NPh.2.2.021010 26158000 PMC4479034

[33] Jaafari N. Canepari M., “Functional coupling of diverse voltage-gated Ca(2+) channels underlies high fidelity of fast dendritic Ca(2+) signals during burst firing,” J. Physiol. 594, 967–983 (2016). JPHYA7 0022-3751 10.1113/JP271830 26634988 PMC4753268

[34] Ait Ouares K. Canepari M., “The origin of physiological local mGluR1 supralinear Ca2+ signals in cerebellar Purkinje neurons,” J. Neurosci. 40, 1795–1809 (2020). JNRSDS 0270-6474 10.1523/JNEUROSCI.2406-19.2020 31969470 PMC7046445

[35] Blömer L. A. et al., “Kinetics and functional consequences of BK channels activation by N-type Ca2+ channels in the dendrite of mouse neocortical layer-5 pyramidal neurons,” Front. Cell. Neurosci. 18, 1353895 (2024). 10.3389/fncel.2024.1353895 38419657 PMC10899506

[36] Yan P. et al., “Palette of fluorinated voltage-sensitive hemicyanine dyes,” Proc. Natl. Acad. Sci. U. S. A. 109, 20443–20448 (2012). 10.1073/pnas.1214850109 23169660 PMC3528613

[37] Zhang H. et al., “Optical mapping of cardiac electromechanics in beating in vivo hearts,” Biophys. J. 122, 4207–4219 (2023). BIOJAU 0006-3495 10.1016/j.bpj.2023.09.017 37775969 PMC10645561

[38] Antic S. D. et al., “ElectroFluor voltage-sensitive dyes: comprehensive analysis of wavelength-dependent sensitivity and cross-channel bleed-through,” J. Biophotonics 18, e70008 (2025). 10.1002/jbio.70008 40103315 PMC12284816

[39] Abbas F. et al., “Neuronal imaging at 8-bit depth to combine high spatial and high temporal resolution with acquisition rates up to 40 kHz,” J. Biophotonics 18, e202400513 (2025). 10.1002/jbio.202400513 39791263 PMC11884961

[40] Savitzky A. Golay M. J. E., “Smoothing and differentiation of data by simplified least squares procedures,” Anal. Chem. 36, 1627–1639 (1964). ANCHAM 0003-2700 10.1021/ac60214a047

[41] Lock J. T. Parker I. Smith I. F., “A comparison of fluorescent ${\mathrm{Ca}}^{2+}$ indicators for imaging local ${\mathrm{Ca}}^{2+}$ signals in cultured cells,” Cell Calc. 58, 638–648 (2015). CECADV 0143-4160 10.1016/j.ceca.2015.10.003 PMC4658286 26572560

[42] Tada M. et al., “A highly sensitive fluorescent indicator dye for calcium imaging of neural activity in vitro and in vivo,” Eur. J. Neurosci. 39, 1720–1728 (2014). EJONEI 0953-816X 10.1111/ejn.12476 24405482 PMC4232931

[43] Liao J. et al., “A novel ${\mathrm{Ca}}^{2+}$ indicator for long-term tracking of intracellular calcium flux,” Biotechniques 70, 271–277 (2021). BTNQDO 0736-6205 10.2144/btn-2020-0161 34000816

[44] Creager R. Dunwiddie T. Lynch G., “Paired-pulse and frequency facilitation in the CA1 region of the in vitro rat hippocampus,” J. Physiol. 299, 409–424 (1980). JPHYA7 0022-3751 10.1113/jphysiol.1980.sp013133 7381775 PMC1279233

[r45] Leung L. S. Fu X. W., “Factors affecting paired-pulse facilitation in hippocampal CA1 neurons in vitro,” Brain Res. 650, 75–84 (1994). BRREAP 0006-8993 10.1016/0006-8993(94)90209-7 7953680

[46] Dumas T. C. Foster T. C., “Developmental increase in CA3-CA1 presynaptic function in the hippocampal slice,” J. Neurophysiol. 73, 1821–1828 (1995). JONEA4 0022-3077 10.1152/jn.1995.73.5.1821 7623083

[47] Quicke P. Barnes S. J. Knöpfel T., “Imaging of brain slices with a genetically encoded voltage indicator,” Methods Mol. Biol. 563, 73–84 (2017). 10.1007/978-1-4939-6810-7_5 PMC5939932 28324602

[r48] Nakajima R. Baker B. J., “Mapping of excitatory and inhibitory postsynaptic potentials of neuronal populations in hippocampal slices using the GEVI, ArcLight,” J. Phys. D Appl. Phys. 51, 504003 (2018). 10.1088/1361-6463/aae2e3 30739956 PMC6366634

[49] Milicevic K. D. et al., “Imaging of evoked cortical depolarizations using either ASAP2s, or chi-VSFP, or Di-4-Anepps, or autofluorescence optical signals,” J. Integr. Neurosci. 22, 160 (2023). 10.31083/j.jin2206160 38176939

[50] Ohkura M. et al., “Genetically encoded green fluorescent ${\mathrm{Ca}}^{2+}$ indicators with improved detectability for neuronal ${\mathrm{Ca}}^{2+}$ signals,” PLoS One 7, e51286 (2012). POLNCL 1932-6203 10.1371/journal.pone.0051286 23240011 PMC3519846

[51] Badura A. et al., “Fast calcium sensor proteins for monitoring neural activity,” Neurophotonics 1, 025008 (2014). 10.1117/1.NPh.1.2.025008 25558464 PMC4280659

[52] Zhu M. H. et al., “Population imaging discrepancies between a genetically-encoded calcium indicator (GECI) versus a genetically-encoded voltage indicator (GEVI),” Sci. Rep. 11, 5295 (2021). SRCEC3 2045-2322 10.1038/s41598-021-84651-6 33674659 PMC7935943

[53] Vogt K. E. et al., “Combining membrane potential imaging with L-glutamate or GABA photorelease,” PLoS One 6, e24911 (2011). POLNCL 1932-6203 10.1371/journal.pone.0024911 22022367 PMC3191132

[54] Willadt S. et al., “Combined optogenetics and voltage sensitive dye imaging at single cell resolution,” Front. Cell. Neurosci. 8, 311 (2014). 10.3389/fncel.2014.00311 25339864 PMC4189389

[55] Milicevic K. D. et al., “Multimodal optical imaging combining voltage-sensitive dye ElectroFluor630 with genetically encoded calcium, glutamate, or voltage indicators,” Neurophotonics 13(S2), S23207 (2026). 10.1117/1.NPh.13.S2.S23207 PMC13233024 42245433

