# Supplementary material for: Combining membrane potential and calcium imaging in brain slices using the voltage-sensitive dye ElectroFluor630 and the calcium indicator Calbryte520
Source: Neurophotonics. 2026 May 13;13(Suppl 2):S23206. doi: 10.1117/1.NPh.13.S2.S23206 (PMC13167447; doi:10.1117/1.NPh.13.S2.S23206)
Supplement: Supplementary file 1 [file NPh_013_S23206_SD001.pdf]

	ROI1 (stim)			***ROI2 ~200 µm from stim***						***ROI3 ~400 µm from stim***					
	Ca^{2+}			***Ca^{2+}***			***EPSP***			***Ca^{2+}***			***EPSP***		
	p-pR	***Δt (ms)***		***p-pR***	***Δt (ms)***		***p-pR***	***Δt (ms)***		***p-pR***	***Δt (ms)***		***p-pR***	***Δt (ms)***	
slice1	0.98	7.2	6.2	1.07	9.6	8.6	1.12	4	4	1.36	9.8	10	1.31	5.6	5.8
slice2	0.95	6.8	6.2	1.04	8.6	8.6	1.19	3.8	3.8	1.21	11.4	9.2	1.15	5	4.6
slice3	0.98	6.2	5.6	0.97	7.4	6.6	1.63	3.8	3.8	0.99	8.6	7.8	1.75	4.8	4.8
slice4	0.96	6.4	6	0.93	9	8.6	1.10	5.2	5	1.01	11.8	13.0	1.76	5.4	5.8
slice5	1.04	8.4	7.8	1.15	10.2	9.6	1.21	3.8	5.0	1.31	10.6	9.6	1.22	4	5.4
slice6	1.12	6.2	5.4	0.93	8.6	7.2	1.38	5.6	5	1.08	10.2	9	1.70	6	5.2
slice7	1.12	5.8	6.8	0.99	6.8	7.6	1.08	3.2	4	0.99	9.4	7.4	1.33	5.2	5.2
slice8	0.94	6.6	7	1.29	9.2	10.8	1.38	4.4	5	1.48	10.6	9.8	1.5	5.4	5.6
slice9	1.00	6.2	5.2	1.44	6.6	7.4	2.22	3.6	3.8	1.31	9.8	10	2.56	4.4	5.2
slice10	0.99	7	6.6	1.35	9	9.8	1.39	4.6	5	1.53	10.2	10	1.5	5.4	5.6
slice11	0.92	6.6	7	1.11	8.2	10.8	1.43	3.8	4	1.35	8.4	9.2	1.80	4.2	4.4
slice12	0.94	6.6	6.8	1.01	8	8.2	1.8	3.2	3.4	1.05	9.4	9.8	1.84	3.6	4

Table S1 Values of paired-pulse ratios (p-pR) of EPSPs and Ca^{2+} transients and the delays between the spike and signals maxima (Δt) from slices where combined EF-630 and Calbrite520 imaging was performed.
